# Synthesis of New Type Polymers by Quasi-Living Atom Transfer Radical Polymerization

**DOI:** 10.3390/polym14142795

**Published:** 2022-07-08

**Authors:** Gergely Illés, Csaba Németh, Karina Ilona Hidas, József Surányi, Adrienn Tóth, Ferenc Pajor, Péter Póti

**Affiliations:** 1 Institute of Animal Sciences, Hungarian University of Agriculture and Life Sciences, Páter Károly 1, 2100 Gödöllő, Hungary; pajor.ferenc@uni-mate.hu (F.P.); poti.peter@uni-mate.hu (P.P.); 2 Capriovus Ltd., 2317 Szigetcsép, Hungary; nemeth.csaba@capriovus.hu; 3 Institute of Food Science and Technology, Hungarian University of Agriculture and Life Sciences, Villányi 29–43, 1118 Budapest, Hungary; hidaskarina@gmail.com (K.I.H.); suranyi.jozsef@uni-mate.hu (J.S.); toth.adrienn@uni-mate.hu (A.T.)

**Keywords:** star polymer, *n*-butyl acrylate, poly(ethylene glycol), atom transfer radical polymerization

## Abstract

Thanks to the polymer revolution of the 20th century, plastics are now part of our everyday lives. We use plastics as naturally as if they had always been an integral part of our lives. However, in the recent past, we were still predominantly using wood, metal, and glass objects, which were replaced by plastic products at an explosive rate. In many cases, this replacement has resulted in products with better physical, chemical, or biological properties. The changeover was too rapid, and the consequences were not recognized in time. This is evidenced by the huge scale of plastic pollution worldwide today. It is therefore in the interests of the future of both humans and animals that we must pay particular attention to the direct and indirect environmental impact of plastics introduced in animal husbandry. Starting from the tetrafunctional initiator produced as the first step of my work, poly(*n*-butyll acrylate) star polymers of different molecular weights were synthesized by atom transfer radical polymerization, using the so-called “core first” method. The bromine chain end of the produced star polymers was replaced by an azide group using a substitution reaction. Propalgyl telechelic PEGs were synthesized as a result of lattice end modification of poly(ethylene glycol) with different molecular weights. The azidated star polymers were connected with propalgyl telechelic PEGs using Huisgen’s “click” chemical process, and as a result of the “click” connection, amphiphilic polymer networks with several different structures were obtained.

## 1. Introduction

In the first half of the 20th century, the emergence of synthetic polymers caused a revolutionary change. After Baekeland’s patent for polycondensation polymer in 1907 [1], the spread of plastics began to develop rapidly. The discovery in 1922 [2] by the German chemist Hermann Staudinger that many organic substances, such as caoutchouc, are made up of very long molecular chains of macromolecules, contributed greatly to this explosive development. His discovery earned him the Nobel Prize in 1953 [3]. One of the special types of polymers, amphilic polymer co-networks, was published for the first time independently by Kennedy [4] and Stadler [5,6] in 1988. The latter studies have relied heavily on living anionic polymerization [7] and quasi-living cationic polymerization [8]. The presence of polymers is common in our environment, and they are found in the pharmaceutical industry, electrical engineering, nanotechnology, information technology, transport, biotechnology, the construction industry, environmental protection, energy production, and the military industry. This wide range of uses has resulted in an increase in the world’s annual production of plastics from 1 million tonnes in 1950 to 360 million tonnes today [9].

Over the past few years, interest in amphiphilic conetworks has grown internationally. Amphiphilic conetworks are materials with special chemical and physical properties, formed by covalently coupled immiscible hydrophilic and hydrophobic polymer chains. Due to their amphiphilic nature, they swell well in both apolar and polar solvents, while retaining their microstructure. They can be biocompatible when built up from suitable components and some of them can show “intelligent” behavior, i.e., they can change their structure reversibly in response to environmental influences (temperature changes, pH changes, ionic strength changes). In addition to nanotechnological applications, amphiphilic conetworks can also be used for the production of implants, controlled drug carriers, and tissue substitutes due to their tissue-friendly properties. In addition, cross-linked biodegradable materials such as polycaprolactone-based amphiphilic materials (APCN) can be prepared from them [10,11,12,13,14].

**Figure 1:**
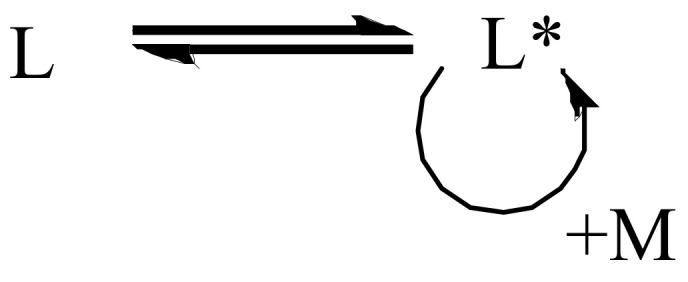
General mechanism of quasi-living polymerization.

In quasi-living polymerizations, the initiated chain grows as long as there is a monomer in the system. In quasi-living polymerizations, chain transfer and irreversible chain termination do not occur. However, reversible chain transfer and chain closure can occur, as the terminated chain (L) can be converted into a chain capable of growth (L*) in the presence of monomer (M). The mechanism of quasi-living polymerization is shown in Figure 1.

If the chain growth takes place via a radical intermediate, we talk about radical polymerization [15]. Radical polymerization has four elementary steps: initiation, propagation, termination, and chain transfer [16]. During initiation, radicals are formed which are attached to monomer molecules by an addition reaction. During propagation, radicals are added to new monomer molecules, increasing the length of the chain. Chain termination occurs when two free radicals link together to form a chain that cannot grow. In chain transfer, the ability to grow is transferred from the chain-carrying radical to another radical.

Polymerizations that have the characteristics of both quasi-living polymerizations and radical polymerizations are called quasi-living radical polymerizations. One type of quasi-living radical polymerization is atom transfer radical polymerization (ATRP) [17]. It was discovered at the end of the last century by Wang, Matyjaszewski, and Sawamoto [18,19]. After its appearance, it spread rapidly throughout the world and is still widely used today [20]. This is due to the large number of monomers that can be polymerized and the wide range of reaction conditions that can be applied.

**Figure 2:**
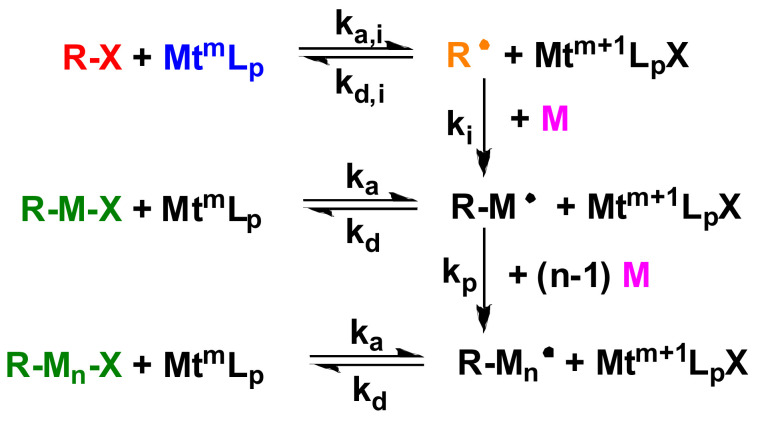
Mechanism of atom transfer radical polymerization (ATRP).

In atom transfer radical polymerization, transition metal catalysis provides the balance between inactive and active chains. The mechanism of ATRP is illustrated in Figure 2, where R–X is the initiator molecule, X is the cleavable halogen atom (Cl or Br) attached to the initiator, Mt^m^L_p_ is the transition metal complex where m is the oxidation number of the transition metal, R۰ is the active free radical formed from the initiator, M is the monomer molecule with an unsaturated double bond, R–M–X is the polymer chain unable to grow, and R–M۰ is the active speciation capable of growth.

The first step in ATRP is the redox reaction of the halogen-containing initiator and the transition metal complex. In the first step, the homolysis of the initiator molecule leads to the detachment of the halogen atom and the formation of a reactive free radical. The oxidation number of the transition metal complex is increased by one because the detached halogen atom links to it. The reactive free radical then reacts with a monomer molecule, and the halogen atom is transferred from the transition metal complex back to the active species capable of further growth, while a polymer chain terminated by a halogen atom incapable of growth is formed. This process continues until there are no free monomer units. After that, the chain ends with additional growth-capable X halogen atoms.

Various initiators can be used in ATRP. These can be mono-, bi-, tri-, or polyfunctional. In the case of polymers produced by ATRP, it is possible to incorporate a functional group at the end of the polymer chain [21] or to replace the halogen group at the end of the chain [22,23]. Most commonly, copper, ruthenium, nickel, iron, molybdenum, rhodium, and palladium halides are applied [24,25,26].

In our work, we have coupled two different branches of polymer chemistry, quasi-living atom transfer radical polymerization and click chemistry. In this way, we coupled properly functionalized star polymers of different number average molecular weights with properly functionalized poly(ethylene glycol) segments of different number average molecular weights. From variations of BA star polymers and PEGs, new types of amphiphilic conetworks with different structures were prepared.

## 2. Materials and Methods

In this experiment, two different branches of polymer chemistry are combined. One of these branches is the synthesis of star polymers. At first, a tetrafunctional initiator was prepared. Afterward, star polymers with different number average molecular weights were synthesized by atom transfer radical polymerization using *n*-(butyl acrylate) (BA) and polystyrene monomers. The other branch of polymer chemistry, which is relatively young, was the preparation of amphiphilic polymeric conetworks by “click” chemistry. The two branches were coupled by linking appropriately functionalized star polymers of different number average molecular weights with appropriately functionalized poly(ethylene glycol) (PEG) segments of different number average molecular weights. From variations of star polymers and PEGs, new types of amphiphilic conetworks with different structures were prepared. Elemental analysis was performed to characterize the structure of the amphiphilic conetworks.

### 2.1. Properties of Used Materials

The materials used for cross-linking and their abbreviations are listed in Table S1, while their properties are shown in Table S2. Tables S1 and S2 are given in the Supplementary File S1.

### 2.2. Cleaning of Used Materials

Toluene: Toluene was refluxed under nitrogen for 4 h in the presence of metallic sodium and benzophenone. Then, it was subjected to distillation. After distillation, it was stored in a dark place under nitrogen.

Tetrahydrofuran (THF): THF was kept on lithium aluminum hydride for 24 h. Then, it was refluxed for a few hours. After distillation, it was used immediately.

BA: The butyl acrylate was stirred on CaH_2_ for 24 h and then vacuum distilled under an Ar atmosphere. It was stored under an inert atmosphere until use.

CuBr and CuCl: Mixing of these substances was carried out with glacial acetic acid for 24 h, and then they were filtered through a glass filter under a nitrogen atmosphere. Then, they were washed three times with absolute ethanol, and six times with diethyl ether. After washing, storage was carried out in a dark place.

Styrene: The styrene used for the polymerization was stirred on CaH_2_ for a few hours and then vacuum distilled under Ar.

DMF: The dimethylformamide used as solvent was stirred overnight in CaH_2_ and then vacuum distilled under Ar.

### 2.3. Polymers Synthesis

#### 2.3.1. Synthesis of 1,1,1,1-tetrakis [2′-bromo-2′-methylpropionyloxymethyl] Methane Initiator

**Figure 3:**
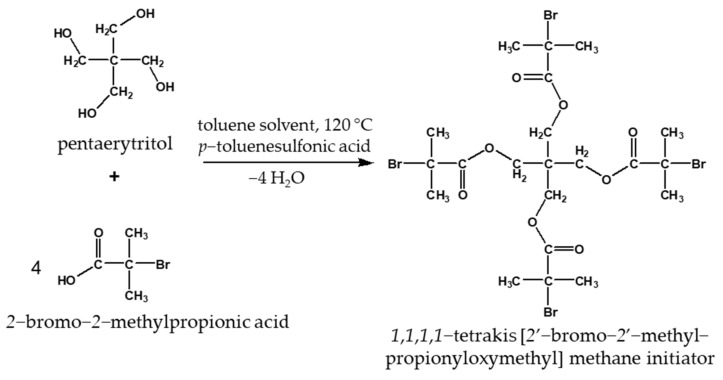
Reaction equation for initiator synthesis.

A tetrafunctional initiator with four functional groups, 1,1,1,1-tetrakis [2′-bromo-2′-methyl propionyloxy] methane (TBMPMM) was prepared. The initiator synthesis was performed in a distillation apparatus. The distillation apparatus was purged with N_2_ gas. Pentaerythritol, α-bromoisobutyric acid, *p*-toluenesulfonic acid, and toluene were measured in a 500 cm^3^ flask according to the method of Kong and Pan [27]. During the azeotropic distillation, a Dean–Stark apparatus was used so that the water formed during the esterification could be easily removed. The reaction was carried out at 110 °C for 3 days, continuous stirring and heating were performed with a heated magnetic stirrer, and a cryostat was used to produce the cooling water. The proportions and exact weights of the materials used are given in Table 1 and the reaction equation is shown in Figure 3.

After stopping the reaction, TBMPMM was extracted first with 5 × 60 cm^3^ of 2% *w*/*w* NaOH solution and then with 3 × 60 cm^3^ of distilled water. The upper toluene phase was then dried over MgSO_4_ overnight. After trapping the water, the MgSO_4_ was filtered through a pleated filter, and the toluene used as solvent was removed on a rotary evaporator. The resulting pale yellow solid was recrystallized from methanol at 40 °C and the precipitated white crystals were dried in a vacuum oven.

#### 2.3.2. Synthesis of Poly (*n*-butyl acrylate) Star Polymers by Quasi-Living Radical Polymerization

Using HMTETA complexing agent, anisole solvent, and *n*-butyll acrylate monomer, two-star polymers with different number average molecular weights were prepared by atomic transfer radical polymerization [28].

**Figure 4:**
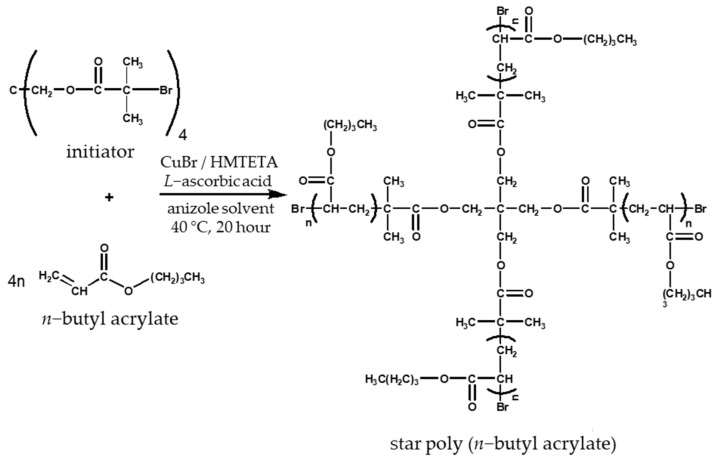
Reaction equation of the star poly (*n*-butyll acrylate) synthesis.

The anisole solvent, TBMPMM initiator, CuBr catalyst, l-ascorbic acid, and *n*-butyll acrylate monomer were weighed in a Schlenk’s vessel. The vessel was sealed with a septum and frozen with dry ice. Argon gas was used to purge the reaction chamber, and then it was removed by vacuum. This deoxygenation process was repeated 5 times. The HMTETA complexing agent was added through the septum using a syringe. The reaction was carried out at 40 °C under argon for 20 h with continuous stirring. The accurately measured amounts of materials used for the syntheses are shown in Table 2 and Table 3. The mechanism of the reaction is illustrated in Figure 4.

The purification process of the star polymers was performed in the next step. The catalyst, the unreacted monomer, and l-ascorbic acid were removed using a column containing neutral alumina and quartz sand. The column was moistened with freshly purified THF, then the anisole solution of the polymer flowed through it, and finally, the column was rinsed with THF. The anisole solvent and the THF used for the column were then removed by rotary evaporation. The pure polymers were dried in an oven at 50 °C for 24 h and stored in a refrigerator until use.

### 2.4. Modification of the Chain End of Star Polymers by a Substitution Reaction

**Figure 5:**
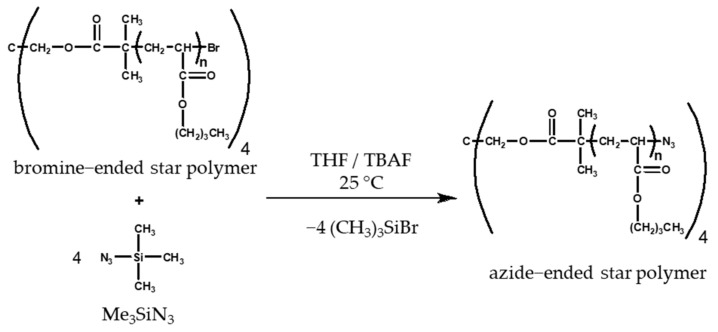
Reaction equation for poly (*n*-butyl acrylate) star polymer end group modification.

The bromine chain end of the prepared four-stranded star polymers was replaced with an azide group by a substitution reaction [29]. The equations of the substitution reaction are shown in Figure 5.

The star polymers were weighed and then dissolved in freshly distilled THF. Me_3_SiN_3_ and TBAF were added to the mixture. The accurate amounts of the substances are shown in Table 4 and Table 5. The solution was stirred for 24 h at room temperature under a nitrogen atmosphere. After completion of the reaction, the modified polymer was precipitated in 10-fold cold methanol, then dried in a vacuum oven overnight and stored in a refrigerator until use.

### 2.5. Production of Propargyl Telechelic Polyethylene Glycol

**Figure 6:**
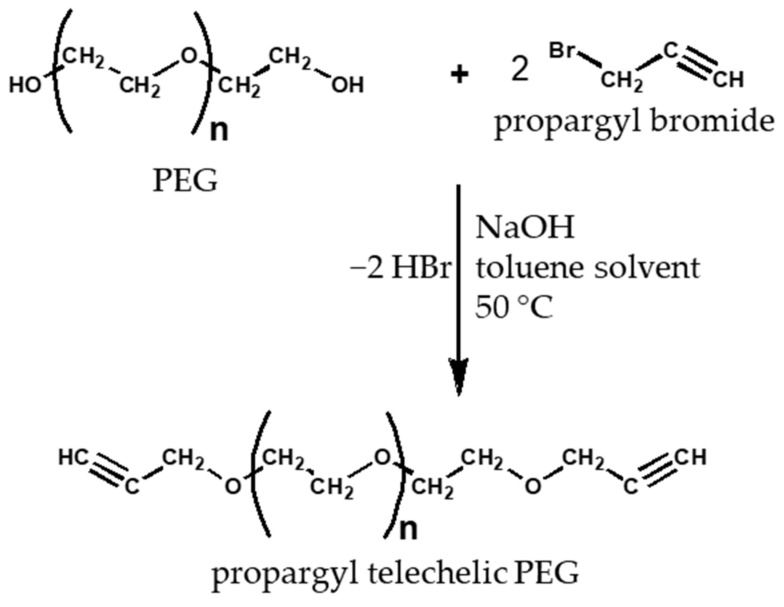
Reaction equation for end-of-chain modification of PEG.

The end groups of commercially available poly (ethylene glycol) with the molar weights of 1500 g/mol (PEG_1500_) and 6000 g/mol (PEG_6000_) were replaced with alkyne functional groups. The poly (ethylene glycol) was weighed, dissolved in toluene, and sodium hydroxide was added to the solution. Propargyl bromide was added to the solution using a syringe. The reaction proceeded at 50 °C for 17.5 h for PEG_1500_ and 24 h for PEG_6000_ [30,31]. The amount of the substances during the end group modification is shown in Table 6 and Table 7 and the equation of the reaction is shown in Figure 6.

After completion of the reaction, the toluene used as a solvent was removed by rotary evaporation. The thick, solvent-free mixture thus obtained was dissolved in 50 cm^3^ of distilled water and then salted out with NaOH. Extraction was performed with 4 × 25 cm^3^ of CH_2_Cl_2_, then the CH_2_Cl_2_ phase was collected, and the mixture was washed with 2 × 10 cm^3^ of distilled water. Drying was carried out over MgSO_4_ overnight and filtering was performed from the desiccant. It was precipitated in cold diethyl ether and dried in an oven. It was stored in a refrigerator until use.

### 2.6. Synthesis of a New Type of Amphiphilic Conetworks

Polymer conetworks were synthesized by a chemical “click” coupling reaction [32] of azide-terminated star polymers and propargyl telechelic PEGs.

**Figure 7:**
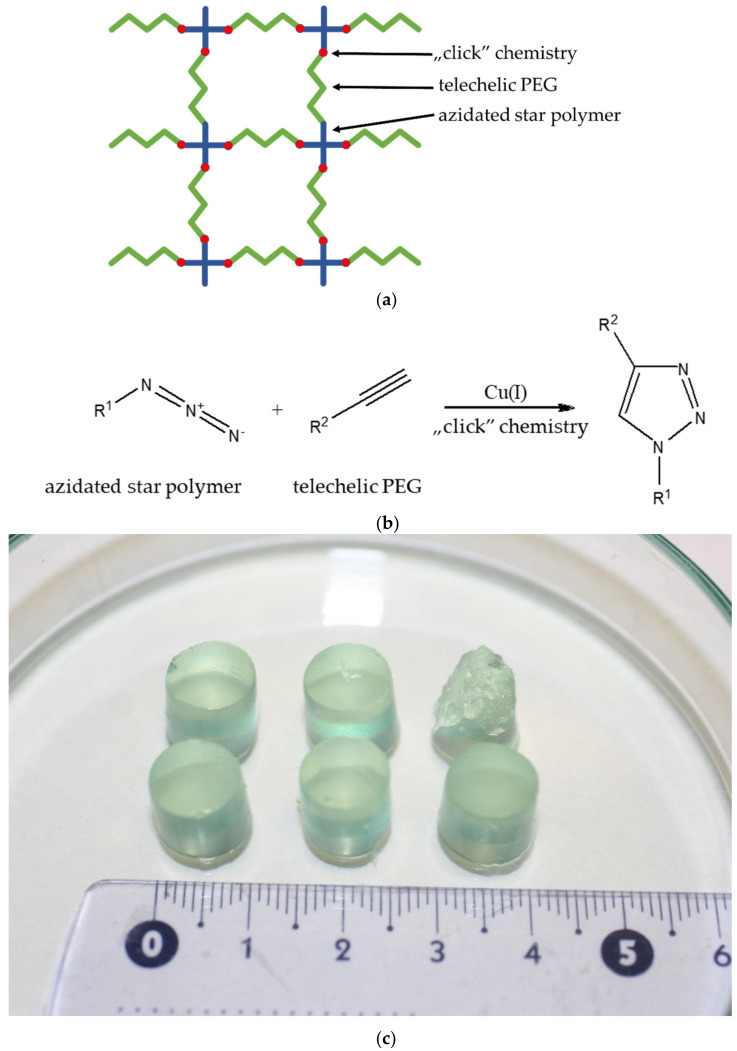
Schematic diagram (**a**) and the “Click” chemistry (**b**) and real image (**c**) of a conetwork generated by a “click” connection.

In the synthesis of *n*-butyll acrylate monomer-containing conetworks, the target star polymer and PEG were dissolved in toluene, and CuCl catalyst and PMDETA complexing agent were added to the solution. Using a Pasteur pipette, the mixture was added to a Teflon tube. By varying the reaction conditions and by combinations of star polymers and PEGs with different number average molecular weights, amphiphilic polymer conetworks with different compositions were prepared. The materials used in the cross-linking processes and the actual reaction conditions are presented in Table 8, Table 9, Table 10 and Table 11 and the schematic diagram of a conetwork generated by a “click” connection is shown in Figure 7.

Completion of cross-linking was checked by swelling in THF, as THF dissolves all the components of the conetwork, but does not dissolve the conetwork itself. The cleaning of conetworks was carried out by swelling in four different solvents. Conetworks were swollen overnight in THF, DCM, acetone, and methanol for 14–14 h. Each swelling was followed by drying at room temperature for 10 h. This method can be used to remove uncross-linked PEG and star polymer as well as copper used as a catalyst [33].

### 2.7. Method of Analysis

#### 2.7.1. Gel Permeation Chromatography

Gel permeation chromatography (GPC) is the most widely used method for determining the molecular weight and molecular weight distribution of polymers. The principle of the measurement is that the molecule to be tested is passed through a column containing a porous cross-linked polymer having no affinity for the molecule. Larger molecules, i.e., larger hydrodynamic volumes, can penetrate fewer pores, so they will elute first. The disadvantage of this method is that it is a calibration procedure based on a relative molecular weight determination, in which the elution volume–hydrodynamic volume function is obtained from a calibration curve prepared using narrow molecular weight standards with narrow molecular weight distribution. Based on this, the molecular weight distribution can be calculated, and from this, the number average (M_n_) and weight average (M_w_) molecular weight. Their quotient M_w_/M_n_ is the polydispersity, which shows how wide the molecular weight distribution is.

GPC measurements were performed with a column system (Mixed C) containing 3 connected μ-Styragel columns (Varian). The different pore sizes were 10^5^, 10^4^, 10^3^, 10^2^, 50, and 10 nm, using freshly distilled tetrahydrofuran containing 0.01 g/l of antioxidant as eluent. The elution rate was 1.0 mL/min. A differential refractometer and differential viscometer were used as detectors. The molecular weight distribution was determined using a universal calibration curve. The chromatograms were evaluated with the program of Viscotec.

#### 2.7.2. Nuclear Magnetic Resonance

Nuclear magnetic resonance spectroscopy (NMR) is a widely used structure-determining method today. ^1^H-NMR and ^13^C-NMR analyses were performed on a Gemini-200 device (Varian) at room temperature. For the determination of the proton spectra, 20–40 mg of sample was diluted in approx. 0.6 mL of denatured chloroform and for the carbon spectra, 40 mg of the sample was diluted in the same way. The spectra were evaluated using a program called ACV SpecView.

#### 2.7.3. Mass Spectroscopy

Mass spectrometry is a large-scale analytical method suitable for the qualitative and quantitative determination of both organic and inorganic gaseous substances. Measurement steps are the following: evaporation, ionization/fragmentation, ion acceleration, separation based on *m*/*z* (mass/charge) values, detection. It can be used to determine molecular weight, chemical structure, and functional groups.

#### 2.7.4. Differential Scanning Calorimetry

Differential scanning calorimetry (DSC) is a method for measuring the heat of the first- and second-order phase transition of substances. DSC devices studying the thermal behavior of polymers have two separately heated sample containers. One contains the sample and the other the inert control sample. The temperature control system changes the temperature linearly between two predetermined points and monitors the temperature change between the sample and the reference sample. If the temperature of the sample decreases or increases compared to the reference sample as a result of the physical or chemical process, it will heat or cool until the temperature is the same. The amount of energy absorbed or released by the sample can be measured and plotted as a function of the temperature of the reference material.

Upon melting, an endothermic peak is obtained, where the peak temperature is considered to be the transition temperature. During the crystallization, the heat released is measured, in which case the temperature of the peak is considered to be the temperature of the crystallization. During glass transition, the heat capacity changes as a function of temperature; here, the glass transition temperature (T_g_) is the change in the slope of the energy-temperature curve. Below the glass transition temperature, the polymers are brittle (glassy); above that, they are flexible. The glass transition temperatures were determined between −120 and 100 °C in a nitrogen atmosphere at a heating rate of 10 °C/min. I considered the second heating cycle of the samples.

#### 2.7.5. Thermogravimetry

Thermogravimetric analysis (TGA) measurements were performed between 35 and 750 °C in a nitrogen atmosphere at a heating rate of 10 °C/min. During the thermogravimetric measurement, the change in mass of the samples was measured as a function of temperature.

#### 2.7.6. Elemental Analysis

Before the elemental study, samples were prepared, because well-dispersed samples are required for elemental analysis measurements. Therefore, samples were frozen in liquid nitrogen. After that, they were pulverized between two metal plates using a hammer.

The elemental analysis of the new type of amphiphilic polymer conetworks was carried out by a Heraeus CHN-O-RAPID (Heraeus, Hanau, Germany) elementary analyzer. The hydrogen, carbon, and nitrogen content of conetworks was determined in a pure oxygen atmosphere with a CuO catalyst by combustion.

#### 2.7.7. Determination of Swelling Degree

A characteristic property of cross-linked polymers is their swelling. Amphiphilic polymer conetworks can swell in both polar and apolar solvents. In the swelling studies, solvents were used that dissolved and swelled one of the components of the conetworks well, but not the other. The swelling was tested in water and hexane. One of the solvents used was water, which is a good solvent for PEG but does not dissolve BA. The other was hexane, which is a good solvent for BA but not for PEG. During swelling, the amphiphilic conetworks increased in volume and mass. The degree of swelling was determined by gravimetry.

Sample pieces of the same size were cut from the conetworks and were air-dried for a few days and then vacuum-dried for a day. After drying, their weight was measured and the sample pieces were put in the solvents.

The hydrated conetwork was taken off and the mass was weighed after 5, 10, 20, 30, 60, 90, 120, 180, 240, 300, 360 min, 1 and 2 days, and 1 week. The swelling degree was calculated by Equation (1).

(1) $$R=\frac{m_{s}-m_{d}}{m_{d}},$$

where *m_d_* is the dry mass of the conetwork, m_s_ indicates the mass of the conetwork after swelling, and *R* is the swelling degree. The swelling degree is illustrated against time.

## 3. Results

### 3.1. Synthesis of 1,1,1,1-tetrakis [2′-bromo-2′-methylpropionyloxymethyl] Methane Initiator

**Figure 8:**
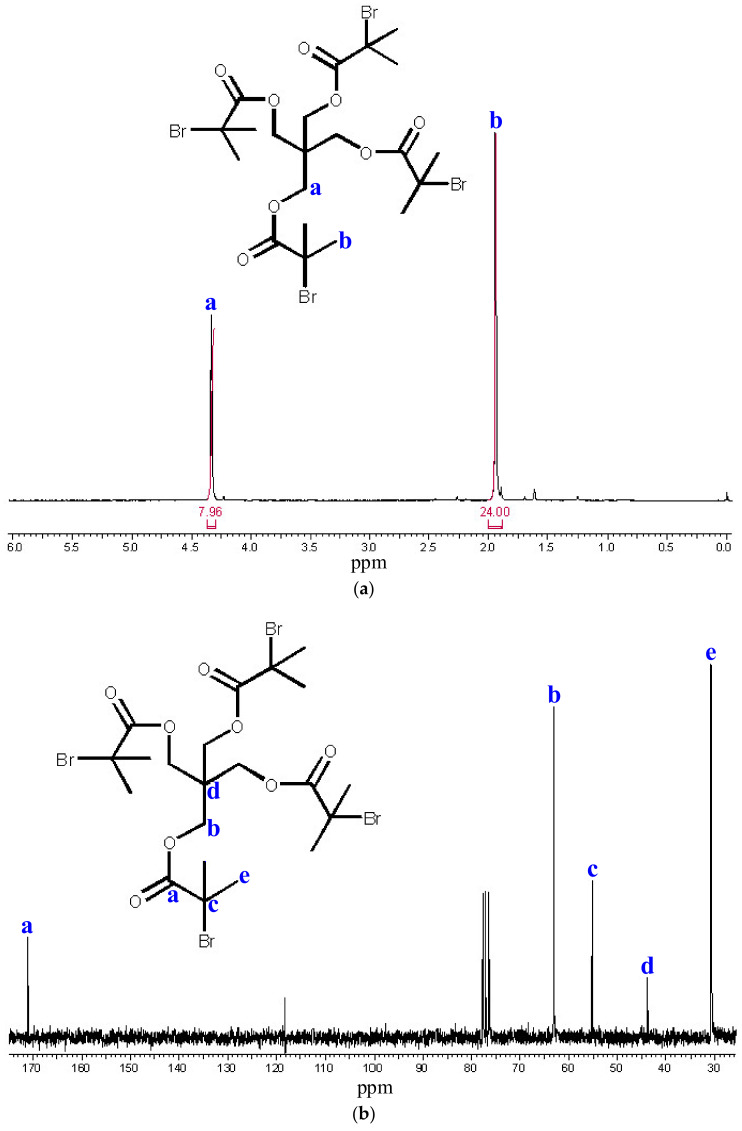
^1^H -NMR (**a**) and ^13^C -NMR (**b**) spectra of the tetrafunctional initiator. The ^1^H -NMR spectrum shows the signals of the hydrogens (**a**) near the ester bond and the signals of the methyl hydrogens (**b**) near the bromine atom.

**Figure 9:**
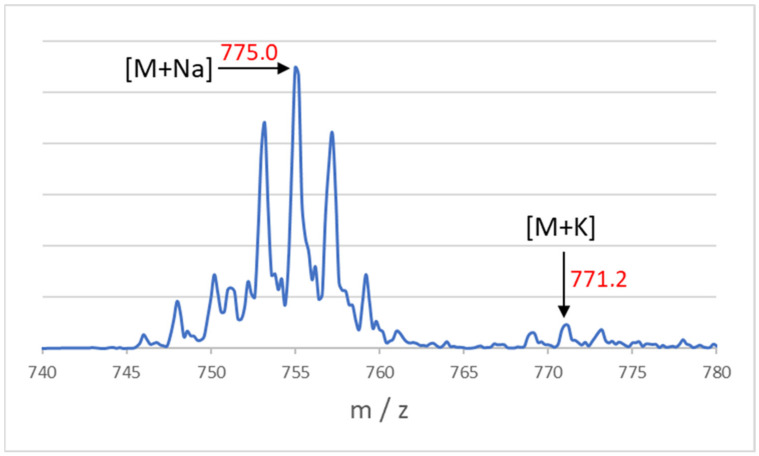
Mass spectrum of the tetrafunctional initiator.

The synthesis of the tetrafunctional initiator forming the core of star polymers was carried out in a nitrogen atmosphere at 120 °C with continuous stirring for 3 days with a yield of 38.87%. After purification and drying of the product, 14.32 g of white crystals was obtained. The ^1^H-NMR spectrum (Figure 8) shows the signals of the hydrogens (a) near the ester bond at 4.33 ppm and the signals of the methyl hydrogens (b) near the bromine atom at 1.94 ppm. The ^13^C-NMR spectrum and the associated structure are shown in Figure 8. Based on mass spectroscopic measurements (Figure 9), the molecular weight of the tetrafunctional initiator is 732 *m*/*z*. These measurement results reflect the efficiency of the synthesis.

### 3.2. Synthesis of Star *n*-butyll acrylate Polymers by Atomic Radical Polymerization

During the star polymer synthesis, *n*-butyll acrylate monomer units were coupled to the prepared tetrafunctional initiator by atomic transfer radical polymerization. As a result of the polymer synthesis of the “core first” method, we obtained four-arm star polymers. This study aimed to cross-link these macromolecules by “click” chemical coupling of azide and alkyne end groups, so the replacement of the bromide chain ends of the polymers was carried out with azide groups by a substitution reaction.

**Figure 10:**
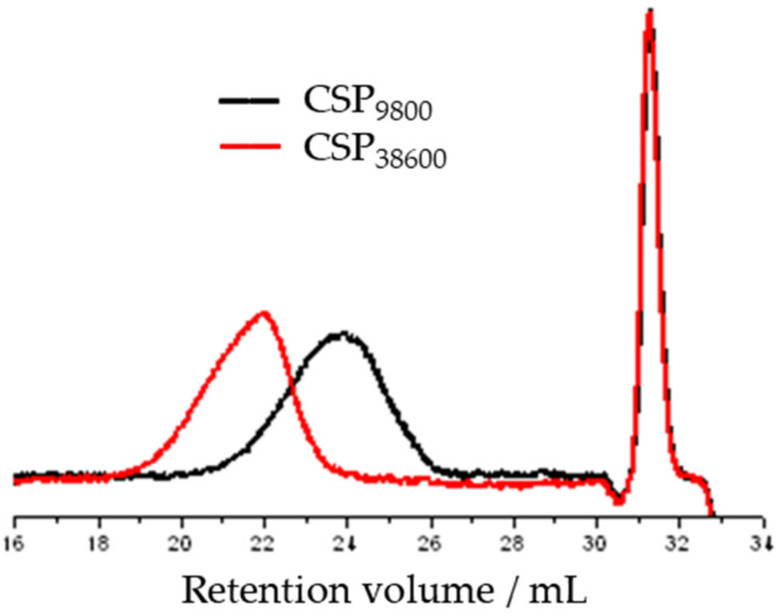
GPC chromatograms of poly(*n*-butyl acrylate) star polymers prepared by ATRP.

**Figure 11:**
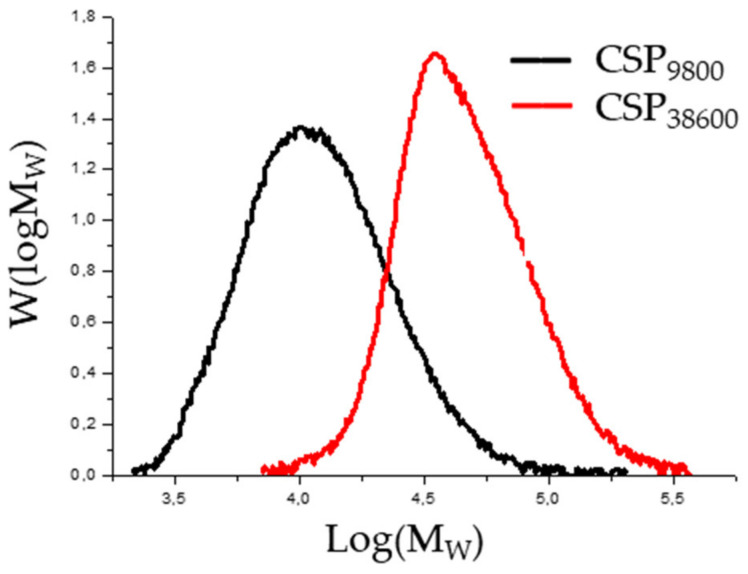
The molecular weight distribution curve of poly(*n*-butyl acrylate) star polymers prepared by ATRP.

The goal of this study was to produce a star polymer with a molecular weight of 5000 g/mol and 25,000 g/mol. The results of the gel permeation chromatographic analysis after polymerization showed that the number average molecular weight of the resulting narrowly distributed macromolecules differed from the design (Figure 10 and Figure 11). One became 9800 instead of 5000 and the other became 38,600 instead of 25,000. The data measured during the GPC test, the calculated yield, and the polymerization degree are summarized in Table 12.

**Figure 12:**
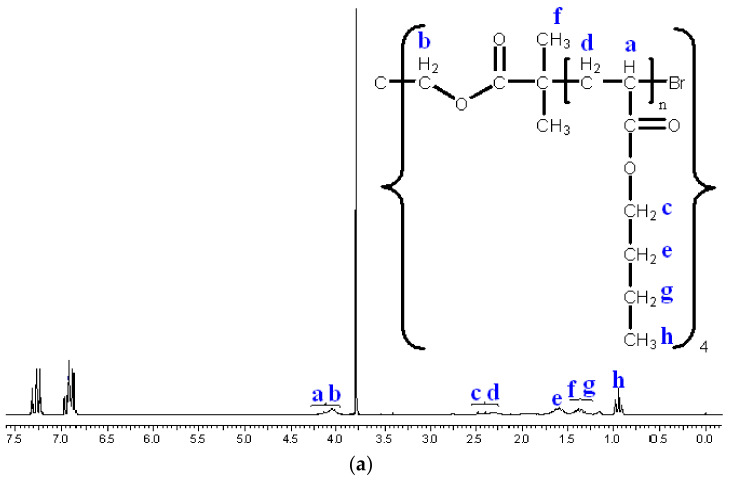

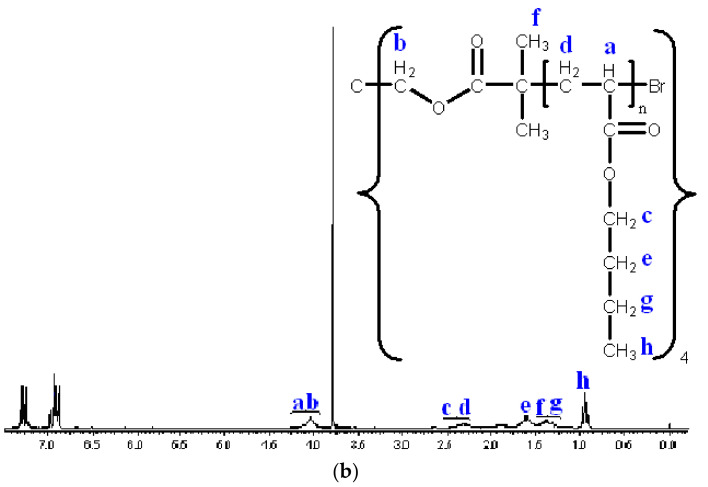
^1^H-NMR spectra of 9800 (**a**) and 38600 (**b**) average molecular weight star polymers. The ^1^H-NMR spectra show the methyl (**f**,**h**) and methylene (**b**,**c**,**d**,**e**,**g**) protons of the star polymers, and the methine proton (**a**) next to the bromine.

The ^1^H-NMR images taken after the synthesis (Figure 12) show the methyl (f, h) and methylene (b, c, d, e, g) protons of the star polymers, and the methine proton (a) next to the bromine. The results of GPC and ^1^H-NMR studies demonstrate the success of ATRP and star polymer synthesis.

### 3.3. Modification of the Chain End of Star Polymers by a Substitution Reaction

**Figure 13:**
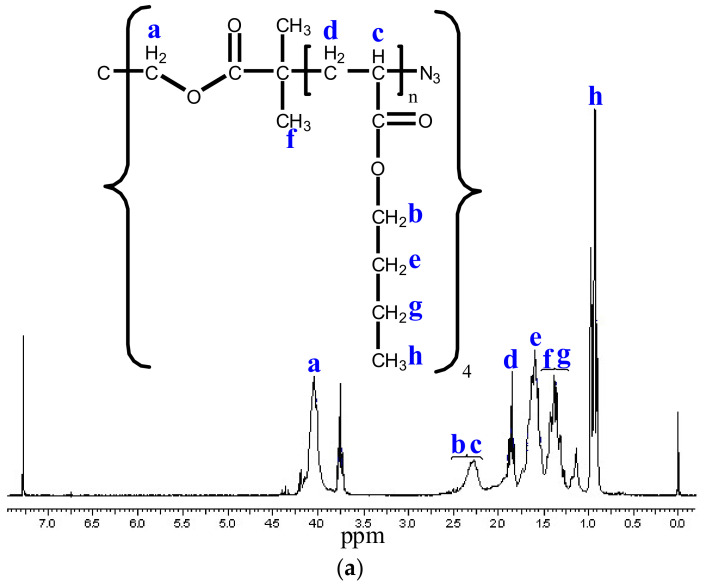

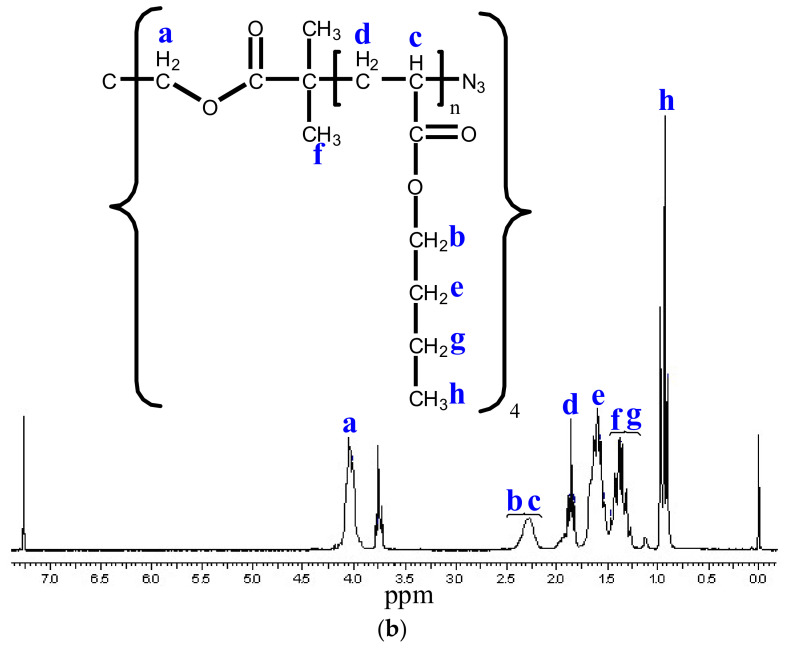
^1^H-NMR spectra of azidated poly (*n*-butyl acrylate) star polymers with the average molecular weight of 9800 (**a**) and 38,600 (**b**). The ^1^H-NMR spectra show the signals of methine protons (**c**) next to the methyl (**h**,**f**), methylene (**a**,**b**,**d**,**e**,**g**).

The Br chain end of the star polymers was replaced by azide groups by a substitution reaction. The ^1^H-NMR spectra (Figure 13) show the signals of methine protons (c) next to the methyl (h, f), methylene (a, b, d, e, g), and azide groups. From these results, we concluded that stable azide end groups were formed during successful chain-end modification.

### 3.4. Functionalization of Poly (Ethylene Glycol)

**Figure 14:**
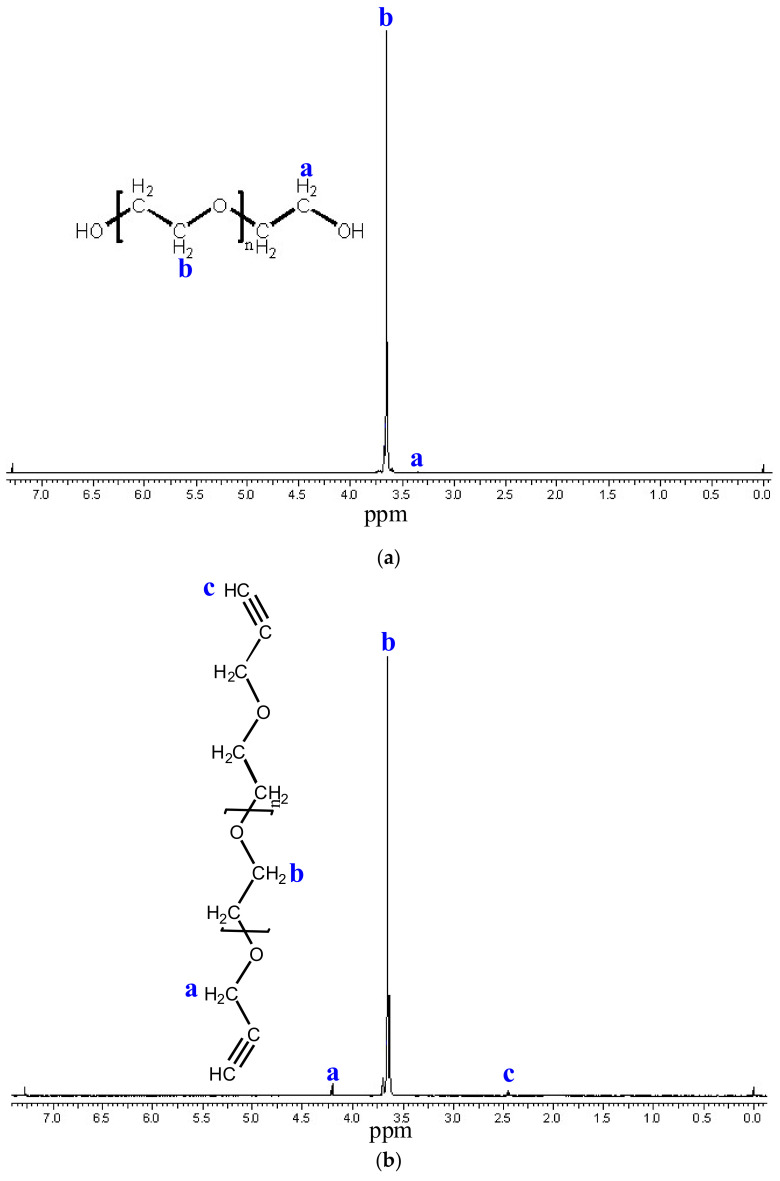
^1^H NMR spectra of PEG_1500_ (**a**) and PEG_1500_alkin_ (**b**). The ^1^H-NMR spectra show the methylene proton (**a**,**b**) and methine proton (**c**).

**Figure 15:**
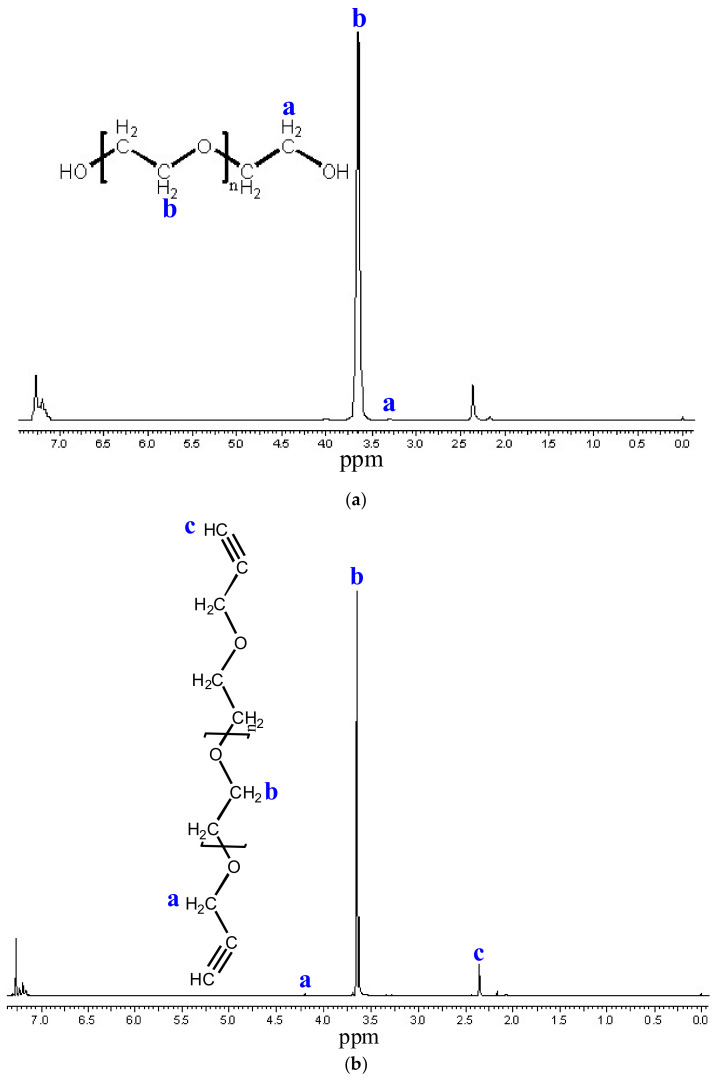
^1^H NMR spectra of PEG_6000_ (**a**) and PEG_6000_alkin_ (**b**). The ^1^H-NMR spectra show the methylene proton (**a**,**b**) and methine proton (**c**).

By modifying the OH end groups of bifunctional PEG1500 (M_n_ = 1500) and PEG_6000_ (M_n_ = 6000), telechelic bialkin end group PEGs were prepared. In both cases, a white waxy substance was formed. During the purification of PEG_1500_, the extraction step was misestimated (complete separation of the two phases was not expected), so the yield was 51%. In the case of PEG_6000_, the error described above was eliminated, so the yield increased to 67%. The formation of alkyne end groups was examined by ^1^H-NMR. For both PEGs, the spectra show that the methylene proton (a) and methine proton (c) characteristics of the alkyne end group appear (Figure 14 and Figure 15). It can be concluded that the formation of the bialkin end group was successful.

**Figure 16:**
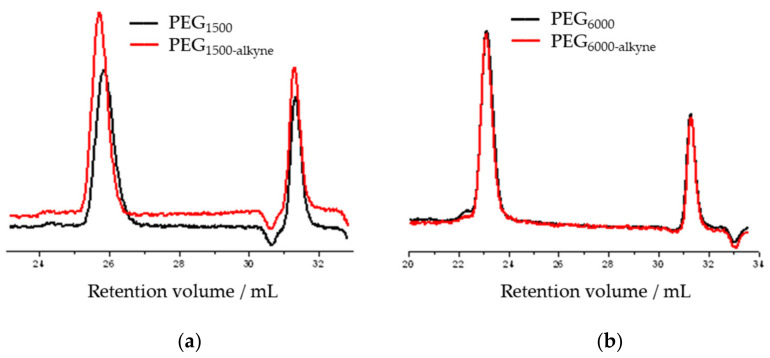
GPC chromatogram of hydroxy and alkyne telelectric PEG_1500_ (**a**) and PEG_6000_ (**b**).

**Figure 17:**
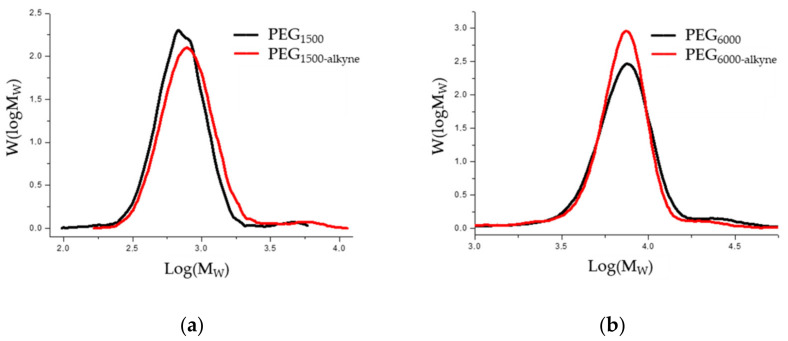
Molecular weight distribution curves of hydroxy and alkyne telecom PEG_1500_ (**a**) and PEG_6000_ (**b**).

The chromatograms of the gel permeation chromatographic analysis are shown in Figure 16. The molecular weight distributions calculated from the chromatograms are shown in Figure 17. The results of the GPC analysis show that narrowly distributed telechelic polymers were formed in both PEG_1500_ and PEG_6000_. The introduction of the alkyne end group did not significantly shift the molecular weight distribution, suggesting that no chain closure or coupling side reactions occurred. The summary of the data is provided in Table 13.

### 3.5. Synthesis of a New Type of Amphiphilic Conetworks

**Figure 18:**
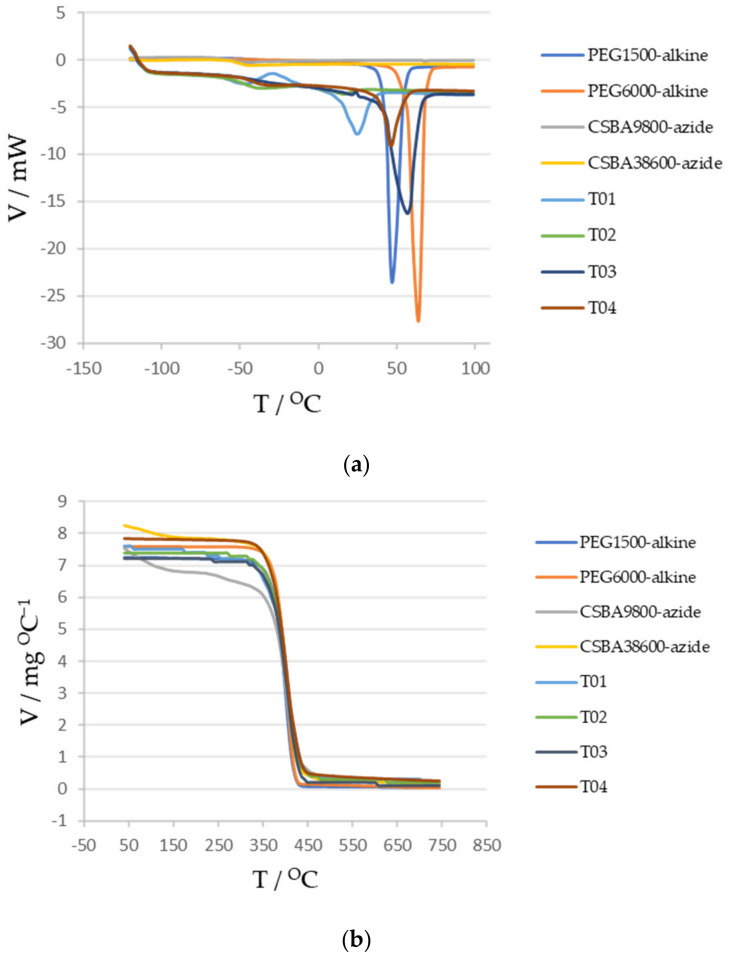
DSC curves (**a**) and TG curves (**b**) of poly (*n*-butyl acrylate) conetworks.

The chain-end modified, azidated poly(*n*-butyll acrylate) and polystyrene star polymers were cross-linked by click coupling reaction with propargyl telechelic PEGs (Figure 18). Table 14 shows the properties of the new types of amphiphilic conetworks.

### 3.6. Differential Scanning Calorimetry and Thermogravimetry

The glass transition temperature of the conetworks was determined by differential scanning calorimetry (DSC) measurements. From the obtained T_g_ data, it is possible to deduce the thermal properties of the investigated conetworks (Figure 18). Thermogravimetric measurements showed high weight loss in all conetworks around 300 °C.

### 3.7. Swelling of Polymer Conetworks

**Figure 19:**
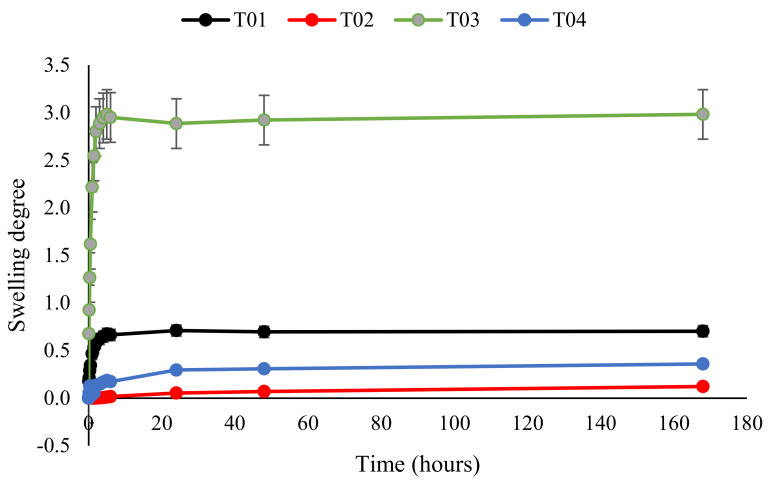
The degree of swelling of butyl acrylate conetworks as a function of time when swollen in water.

**Figure 20:**
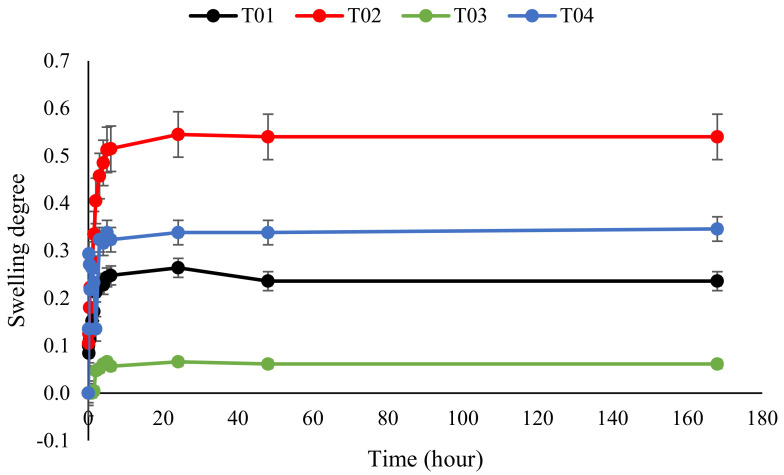
The degree of swelling of butyl acrylate conetworks as a function of time when swollen in hexane.

The swelling was tested in water and hexane. Swelling agents were chosen based on the fact that they swell only one component of the conetwork. Based on preliminary solubility tests, water dissolves only PEG and hexane dissolves only PBA. The calculated swelling rates are plotted as a function of time (Figure 19 and Figure 20). The plots clearly illustrate that conetworks with higher PEG content swell more in water and conetworks with higher BA content swell more in hexane.

### 3.8. Elemental Analysis

**Figure 21:**
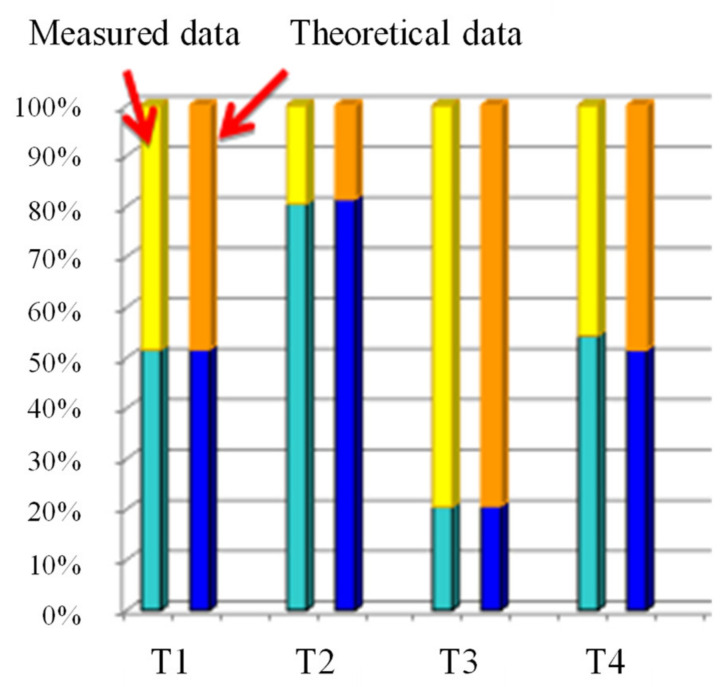
The BA measured data (light blue) and theoretical data (dark blue), and the PEG measured data (yellow) and the PEG theoretical data (orange) content of the conetworks in mol%.

Elemental analysis tests were carried out to determine the composition of the produced conetworks. The carbon and hydrogen, measurement results obtained, and the BA and PEG content calculated from them are presented in Table 15 and Figure 21.

The molar percentages of PEG and BA obtained in the elemental analysis agree with the amount of water taken up by PEG (PEG content) and hexane taken up by BA (BA content) during swelling. Thus, this method can be used to produce amphiphilic conetworks with a well-defined structure.

## 4. Conclusions

The interest in new types of amphiphilic conetworks has increased dramatically in recent years due to their amphiphilic nature, nanophase morphology, and biocompatibility. The aim of this study was to synthesize polymer conetworks for use in animal husbandry by a new method combining quasi-living atom transfer radical polymerization and “click” reaction. A tetrafunctional initiator was synthesized by an etherification reaction, and new types of amphiphilic conetworks were created by quasi-living atom radical transfer polymerization with the preformed initiator, cross-linked by “click” chemical reaction.

In this study, different polymer conetworks were synthesized by “click” reaction, and at the same time, the suitability of the “click” reaction for the synthesis of new types of amphiphilic polymeric conetworks with different structures was demonstrated. These new materials could potentially have applications in various areas of animal husbandry, such as new types of polymeric flooring with bactericidal, virucidal, and microbicidal properties, created as an improvement to the housing technology of cattle sheds.

## Figures and Tables

**Table 1:** Substances measured during the synthesis of the TBMPMM initiator.

Name	Molar Ratio	Weight (g)	Measured Quantity (g)
Pentaerythritol	5	6.8	6.77
2-Bromo-2-methylpropionic acid	25	42	42.40
*p*-toluenesulfonic acid	1	2	2.03
Toluene (solvent)	-	173	172.98

**Table 2:** Substances measured during the synthesis of 9800 number average molecular weight star polymer.

Name	Molar Ratio	Weight (g)	Measured Quantity (g)
l-ascorbic acid	6	2.1134	2.1510
CuBr	0.4	0.1148	0.1142
HMTETA	0.4	0.5530	0.5530
Initiator	1	1.4642	1.4658
*n*-butyll acrylate	33	8.5358	8.5523
Anisole	80	17.3303	17.3687

**Table 3:** Substances measured during the synthesis of 38,600 number average molecular weight star polymer.

Name	Molar Ratio	Weight (g)	Measured Quantity (g)
l-ascorbic acid	6	0.4227	0.4224
CuBr	0.4	0.0229	0.0264
HMTETA	0.4	0.1106	0.1106
Initiator	1	0.2929	0.2926
*n*-butyll acrylate	190	9.7071	9.7218
Anisole	80	19.7084	19.7475

**Table 4:** Substances measured during modification of CSBA_9800_.

Name	Molar Ratio	Weight (mg)	Volume (mL)	Measured Quantity
Me_3_SiN_3_	20	0.9137	1.043	1.10 mL
TBAF	20	-	7.945	8.00 mL
THF	479	13.82	15.54	15.50 mL
CSBA_9800_	1	3.885	-	3.885 g

**Table 5:** Substances measured during modification of CSBA_38600_.

Name	Molar Ratio	Weight (mg)	Volume (mL)	Measured Quantity
Me_3_SiN_3_	20	0.2392	0.2731	0.28 mL
TBAF	20	-	2.08	2.10 mL
THF	1978	14.26	16.04	16.00 mL
CSBA_38600_	1	4.010	-	4.010 g

**Table 6:** Substances measured during modification of PEG_1500_.

Name	Molar Ratio	Weight (mg)	Volume (mL)	Measured Quantity
Propargyl bromide	20	7.9299	7.2	7.4 mL
NaOH	20	2.6667	-	2.7 g
PEG_1500_	1	5	-	5.0034 g
Toluene solvent	-	21.625	25	25 mL

**Table 7:** Substances measured during modification of CSBA_38600_.

Name	Molar Ratio	Weight (mg)	Volume (mL)	Measured Quantity
Propargyl bromide	20	1.9939	1.86	1.9 mL
NaOH	20	0.6667	-	0.7 g
PEG_6000_	1	5	-	5.0 g
Toluene solvent	-	21.625	25	25 mL

**Table 8:** Substances measured during the reaction of CSBA_9800-azide_ and PEG_1500-alkyne_.

Name	Molar Ratio	Mol (mmol)	Weight (mg)	Volume (mL)	Measured Quantity
CSBA_9800-azide_	1	0.05	500	-	507 mg
PEG_1500-alkyne_	2	0.1	150	-	151 mg
l-ascorbic acid	2	0.1	20	-	20 mg
PMDETA	4	0.2	35	0.042	0.15 mL
CuCl	4	0.2	20	-	20 mg
Toluene	-	-	-	1	-
Reaction conditions	35 °C, 17 h				

**Table 9:** Substances measured during the reaction of CSBA_38600-azide_ and PEG_1500-alkyne_.

Name	Molar Ratio	Mol (mmol)	Weight (mg)	Volume (mL)	Measured Quantity
CSBA_38600-azide_	1	0.013	500	-	507 mg
PEG_1500-alkyne_	2	0.026	39	-	39 mg
l-ascorbic acid	0.2	0.0026	4.6	-	8.8 mg
PMDETA	0.4	0.0052		0.011	0.022 mL
CuCl	0.4	0.0052	5	-	5.3 mg
Toluene	-	-	-	1	-
Reaction conditions	room temperature, 51 h				

**Table 10:** Substances measured during the reaction of CSBA_9800-azide_ and PEG_6000-alkyne_.

Name	Molar Ratio	Mol (mmol)	Weight (mg)	Volume (mL)	Measured Quantity
CSBA_9800-azide_	1	0.05	500	-	500 mg
PEG_6000-alkyne_	2	0.102	612	-	612 mg
l-ascorbic acid	0.2	0.0102	1.8	-	7.4 mg
PMDETA	0.4	0.0204	3.5	0.042	0.06 mL
CuCl	0.4	0.0204	2	-	20 mg
Toluene	-	-	-	2	-
Reaction conditions	room temperature, 20 h				

**Table 11:** Substances measured during the reaction of CSBA_38600-azide_ and PEG_6000-alkyne_.

Name	Molar Ratio	Mol (mmol)	Weight (mg)	Volume (mL)	Measured Quantity
CSBA_38600-azide_	1	0.013	500	-	507 mg
PEG_6000-alkyne_	2	0.026	39	-	39 mg
l-ascorbic acid	0.2	0.0026	4.6	-	8.8 mg
PMDETA	0.4	0.0052	-	0.011	0.022 ml
CuCl	0.4	0.0052	5	-	5.3 mg
Toluene	-	-	-	1	-
Reaction conditions	room temperature, 20 h				

**Table 12:** Data for star polymers synthesized with ATRP.

Sample	M_n_^1^ (g/mol)	M_w_^2^ (g/mol)	M_w_/M_n_^3^	Yield (%)	Polymerization Degree
CSBA_9800_	9800	15,400	1.57	78	18
CSBA_38600_	38,600	55,000	1.42	80	74

^1^ Number average molecular weight, ^2^ weight average molecular weight, ^3^ polydispersity.

**Table 13:** Data on poly (ethylene glycols).

Name	M_n_^1^ (g/mol)	M_w_^2^ (g/mol)	M_w_/M_n_^3^	Yield (%)	Polymerization Degree
PEG_1500_	1408	1760	1.25	-	34
PEG_1500-alkin_	1606	2120	1.32	51	34
PEG_6000_	5590	8450	1.51	-	136
PEG_6000-alkin_	6030	7760	1.29	67	136

^1^ Number average molecular weight, ^2^ weight average molecular weight, ^3^ polydispersity.

**Table 14:** Reaction conditions and physical properties of the amphiphilic conetworks.

Code of the Amphiphilic Conetwork	Components	Temperature (°C)	Reaction Time (°C)	Color	Remark
**T1**	CSBA_9800-azide_ PEG_1500-alkyne_	35	17	Yellow	Rubbery, sticky
**T2**	CSBA_38600-azide_ PEG_1500-alkyne_	25	51	Blue	Rubbery, very sticky
**T3**	CSBA_9800-azide_ PEG_6000-alkyne_	50	45	Pale blue	Solid, easy to cut
**T4**	CSBA_38600-azide_ PEG_6000-alkyne_	50	192	Pale green	Solid, easy to cut

**Table 15:** Carbon and hydrogen concentration of the synthesized amphiphilic conetworks and their calculated BA and PEG content.

Code of the Amphiphilic Conetwork	C (m/m%)	H (m/m%)	BA mol%	PEG mol%
T1	61.1	9.5	51.5	48.5
T2	64.3	9.3	80.4	19.6
T3	58.4	9.3	20.4	79.6
T4	62.7	9.8	54.3	45.7

## Data Availability

The data presented in this study are available on request from the corresponding author.

## Supplementary Materials

The following supporting information can be downloaded at: https://www.mdpi.com/article/10.3390/polym14142795/s1. Table S1: Abbreviations of the used materials. Table S2: Materials used for and their properties.
