# Supplementary material for: Synthesis of New Type Polymers by Quasi-Living Atom Transfer Radical Polymerization
Source: Polymers (Basel). 2022 Jul 8;14(14):2795. doi: 10.3390/polym14142795 (PMC9316120; doi:10.3390/polym14142795)
Supplement: Supplementary file 1 [file polymers-14-02795-s001.zip › polymers-1802448-supplementary-update.pdf]

polymers

Supplementary Materials

Synthesis of New Type Polymers by Quasi-Living Atom Transfer Radical Polymerization

Gergely Illés [1,*], Csaba Németh [2], Karina Ilona Hidas [3], József Surányi [3], Adrienn Tóth [3], Ferenc Pajor [1] and Péter Póti [1]

[1] Institute of Animal Sciences, Hungarian University of Agriculture and Life Sciences, Páter Károly 1., 2100 Gödöllő, Hungary; pajor.ferenc@uni-mate.hu (F.P.); poti.peter@uni-mate.hu (P.P.)
[2] Capriovus Ltd., 2317 Szigetcsép, Hungary; nemeth.csaba@capriovus.hu
[3] Institute of Food Science and Technology, Hungarian University of Agriculture and Life Sciences, Villányi 29-43., 1118 Budapest, Hungary; hidaskarina@gmail.com (K.I.H.); suranyi.jozsef@uni-mate.hu (J.S.); toth.adrienn@uni-mate.hu (A.T.)
* Correspondence: illesger@gmail.com

Table S1. Abbreviations of the used materials

Abbreviation	Name
APCN	Amphiphilic polycaprolactone
ATRP	**A**tom **T**ransfer **R**adical **P**olymerization
BA	n-butyl acrylate
BPY	2,2'-Bipyridil
$CSBA_{9800}$	poly(butyl acrylate) star polymer with number average molecular weight of 9800
$CSBA_{38600}$	poly(butyl acrylate) star polymer with number average molecular weight of 38600
$CSBA_{9800-azide}$	tetra azide poly(butyl acrylate) star polymer with number average molecular weight of 9800
$CSBA_{38600-azide}$	tetra azide poly(butyl acrylate) star polymer with number average molecular weight of 38600
DMF	dimethylformamide
DSC	differential scanning calorimetry
GPC	gel permeation chromatography
HX	hexane
HMTETA	hexamethyltriethylene tetramine
H_2O	distilled water
Me_3SiN_3	azido-trimethyl silane
$MgSO_4$	magnesium sulfate
MS	mass spectroscopy
NaOH	sodium hydroxide
NMR	nuclear magnetic resonance spectroscopy
PEG	poly(ethylene glycol)
PEG_{1500}	poly(ethylene glycol) with number average molecular weight of 1500
PEG_{6000}	poly(ethylene glycol) with number average molecular weight of 6000
$PEG_{1500-alkyne}$	propargyl telechelic poly(ethylene glycol) with number average molecular weight of 1500

$PEG_{6000\text{-alkyne}}$	propargyl telechelic poly(ethylene glycol) with number average molecular weight of 6000
PMDETA	pentamethyldiethylenetriamine
TBAF	tetrabutylammonium fluoride
TBMPMM	1,1,1,1-tetrakis [2'-bromo-2'-methylpropionyloxymethyl] methane
TG	thermogravimetry
THF	tetrahydrofuran

Table S2. Materials used for and their properties.

Name	M [1] (g/mol)	Purity	Tb [2] (°C)	Tm [3] (°C)	ϱ [4] (g/cm3)	Producer
Anisole	108.14	99%	154	–37	0.995	Aldrich
α-Bromoisobutyric acid	167.01	98%	198-200	48	-	Aldrich
CuBr	143.45	98%	-	504	4.710	Aldrich
CuCl	98.99	99+%	-	430	4,14	Aldrich
$CSBA_{9800}$	9800	-	-	-	-	-
$CSBA_{38600}$	38600	-	-	-	-	-
$CSBA_{9800\text{-azide}}$	9800	-	-	-	-	-
$CSBA_{38600\text{-azide}}$	38600	-	-	-	-	-
Hexane	86.18	99+%	69	–95	0.655	Aldrich
HMTETA	230.40	97%	-	-	0.847	Aldrich
H_2O	18	-	100	0	-	-
L-ascorbic acid	176.12	99+%	-	193	-	Aldrich
Me_3SiN_3	115.21	95%	92-95	-	0.876	Aldrich
n-butyl acrylate	128.17	99+%	145	-	0.894	Fluka
NaOH	40.01	99%	-	318	-	Reanal
p-toluenesulfonic acid	190.22	98.5%	-	103-106	-	Aldrich
PEG_{1500}	1500	-	-	-	-	Aldrich
PEG_{6000}	6000	-	-	-	-	Fluka
$PEG_{1500\text{-alkyne}}$	1500	-	-	-	-	-
$PEG_{6000\text{-alkyne}}$	6000	-	-	-	-	-
Pentaerythritol	136.15	≥99%	276	253-258	-	Aldrich
PMDETA	173.3	99%	-	-	0,830	Aldrich
Propargyl-bromide	118.96	80%	88-90	-	1.335	Aldrich
TBAF	261.47	-	-	-	0.903	Aldrich
TBMPMM	732.09	-	-	-	-	-
THF	72.11	99%	65-67	-108	0.890	Spektrum 3D
Toluene	92.14	99.9%	110-111	–93	0.864	Aldrich

[1] molar mass, [2] boiling temperature, [3] melting temperature, [4] density
